# Can Genetic Analysis of Putative Blood Alzheimer’s Disease Biomarkers Lead to Identification of Susceptibility Loci?

**DOI:** 10.1371/journal.pone.0142360

**Published:** 2015-12-01

**Authors:** Robert C. Barber, Nicole R. Phillips, Jeffrey L. Tilson, Ryan M. Huebinger, Shantanu J. Shewale, Jessica L. Koenig, Jeffrey S. Mitchel, Sid E. O’Bryant, Stephen C. Waring, Ramon Diaz-Arrastia, Scott Chasse, Kirk C. Wilhelmsen, for the Alzheimer’s Disease Neuroimaging Initiative and the Texas Alzheimer’s Research and Care Consortium

**Affiliations:** 1 Department of Molecular & Medical Genetics, University of North Texas Health Science Center, Fort Worth, Texas, United States of America; 2 Institute for Aging and Alzheimer’s Disease Research, University of North Texas Health Science Center, Fort Worth, Texas, United States of America; 3 Department of Biology, University of Dallas, Dallas, Texas, United States of America; 4 Renaissance Computing Institute, University of North Carolina, Chapel Hill, North Carolina, United States of America; 5 Department of Surgery, University of Texas Southwestern Medical Center, Dallas, Texas, United States of America; 6 Department of Internal Medicine, University of North Texas Health Science Center, Fort Worth, Texas, United States of America; 7 Essentia Institute of Rural Health, Duluth, Minnesota, United States of America; 8 Center for Neuroscience and Regenerative Medicine, Uniformed Services University of the Health Sciences, Rockville, Maryland, United States of America; 9 Department of Genetics, University of North Carolina, Chapel Hill, North Carolina, United States of America; 10 Department of Genetic Medicine, University of North Carolina, Chapel Hill, North Carolina, United States of America; University of Leipzig, GERMANY

## Abstract

Although 24 Alzheimer’s disease (AD) risk loci have been reliably identified, a large portion of the predicted heritability for AD remains unexplained. It is expected that additional loci of small effect will be identified with an increased sample size. However, the cost of a significant increase in Case-Control sample size is prohibitive. The current study tests whether exploring the genetic basis of endophenotypes, in this case based on putative blood biomarkers for AD, can accelerate the identification of susceptibility loci using modest sample sizes. Each endophenotype was used as the outcome variable in an independent GWAS. Endophenotypes were based on circulating concentrations of proteins that contributed significantly to a published blood-based predictive algorithm for AD. Endophenotypes included Monocyte Chemoattractant Protein 1 (MCP1), Vascular Cell Adhesion Molecule 1 (VCAM1), Pancreatic Polypeptide (PP), Beta2 Microglobulin (B2M), Factor VII (F7), Adiponectin (ADN) and Tenascin C (TN-C). Across the seven endophenotypes, 47 SNPs were associated with outcome with a p-value ≤1x10^-7^. Each signal was further characterized with respect to known genetic loci associated with AD. Signals for several endophenotypes were observed in the vicinity of CR1, MS4A6A/MS4A4E, PICALM, CLU, and PTK2B. The strongest signal was observed in association with Factor VII levels and was located within the F7 gene. Additional signals were observed in MAP3K13, ZNF320, ATP9B and TREM1. Conditional regression analyses suggested that the SNPs contributed to variation in protein concentration independent of AD status. The identification of two putatively novel AD loci (in the Factor VII and ATP9B genes), which have not been located in previous studies despite massive sample sizes, highlights the benefits of an endophenotypic approach for resolving the genetic basis for complex diseases. The coincidence of several of the endophenotypic signals with known AD loci may point to novel genetic interactions and should be further investigated.

## Introduction

All of the common loci that have been linked to late onset Alzheimer’s disease (AD) other than *APOE* have small effect sizes and a large portion of the predicted heritability for AD remains unidentified[1]. A number of explanations and potential sources have been postulated for this missing heritability, which is observed for many complex human diseases. Examples include rare variants with large effect sizes, epistatic interactions between multiple common alleles, inflated heritability statistics and genetic heterogeneity, among others[2].

Another approach to the identification of genes involved in Alzheimer’s disease pathogenesis is to ascertain quantitative endophenotypes that are associated with AD risk and then look for genetic variants that are associated with those endophenotypes. Endophenotypes are intermediate traits that are closer to the underlying molecular mechanism than the complex phenotype, and are in principle more likely to be affected by the genetic variation. Discovering genetic and environmental factors contributing to complex human diseases, as well as the development of effective therapies often requires understanding endophenotypes of the disease. For example, discovery of genetic factors contributing to coronary artery disease and the eventual development of effective therapies based on HMG-CoA reductase inhibition was made possible by understanding the endophenotype of hypercholesterolemia[3]. Potential endophenotypes of Alzheimer’s disease include quantitative neuroimaging, such as measures of hippocampal atrophy[4–6], or levels of amyloid or tau proteins in the brain or cerebrospinal fluid (CSF)[7–10]. An additional and still evolving source of AD biomarkers is the pool of circulating proteins in the blood[11–14].

Our objective in this project was to identify the genetic variants that impact concentrations of proteins associated with diagnostic status for Alzheimer’s disease. It was expected that genotypes of some variants would be correlated with protein levels and AD status while others would be correlated with protein levels alone. We used conditional regression analysis to assess the relationship between AD risk, biomarkers, SNPs and non-genetic risk factors.

## Materials and Methods

### Study Cohorts—TARCC and ADNI

TARCC methodologies have been described in detail elsewhere[15]. Criteria for categorizing subjects as probable AD, mild cognitive impairment (MCI) or normal control (NC) are based on neurocognitive evaluations, family and/or caregiver interviews and medical history. NC must have normal psychometric test scores and a clinical dementia rating (CDR) score of 0. MCI subjects are classified based on the Mayo Clinic Alzheimer's Disease Research Criteria[16]. Patients are characterized as probable AD according to the National Institute of Neurological and Communicative Disorders and Stroke (NINCDS) and the Alzheimer’s Disease and Related Disorders Association (ADRDA) criteria[17]. Each participating site that enrolled participants operates with Institutional Review Board (IRB) approval and each of the following IRBs approved this study (University of North Texas Health Science Center IRB, University of Texas Southwestern Medical Center IRB, Baylor College of Medicine IRB, University of Texas Health Science Center at San Antonio IRB, Texas Tech University Health Sciences Center IRB). Written informed consent was obtained for every participant at the site of enrollment. Data that were used in this study as a validation set were obtained from the Alzheimer’s Disease Neuroimaging Initiative (ADNI) (adni.loni.usc.edu). Details of ADNI clinical evaluation and sample characterization are described elsewhere[18, 19]. The primary goal of ADNI has been to test whether serial magnetic resonance imaging (MRI), positron emission tomography (PET), other biological markers, and clinical and neuropsychological assessment can be combined to measure the progression of mild cognitive impairment (MCI) and early Alzheimer’s disease (AD). The Principal Investigator of this initiative is Michael W. Weiner, MD, VA Medical Center and University of California–San Francisco. The initial goal of ADNI was to recruit 800 subjects but ADNI has been followed by ADNI-GO and ADNI-2. A complete listing of ADNI investigators can be found at: http://adni.loni.usc.edu/wp-content/uploads/how_to_apply/ADNI_Acknowledgment_List.pdf. To date these three protocols have recruited over 1500 adults, ages 55 to 90. The follow up duration of each group is specified in the protocols for ADNI-1, ADNI-2 and ADNI-GO. For up-to-date information, see www.adni-info.org. Demographic data for the TARCC and ADNI cohorts are provided in Table 1.

**Table 1:** Demographic characteristics of the Texas Alzheimer’s Research and Care Consortium (TARCC) and the Alzheimer’s Disease Neuroimaging Initiative (ADNI) cohorts.

	NC		MCI		AD	
Variable	TARCC N = 134	ADNI N = 41	TARCC N = 0	ADNI N = 298	TARCC N = 166	ADNI N = 84
Age, Mean (SD)	70 (8.9)	76 (5.8)	-	75 (7.6)	76 (8.5)	76 (8.0)
Years of Education, Mean (SD)	15 (2.6)	16 (2.7)	-	16 (2.9)	14 (3.3)	15 (3.1)
Race, N (%)			-			
White	131 (97.8)	41 (100)	-	297 (99.7)	165 (99.4)	84 (100)
Other*	3 (2.2)	0 (0.0)	-	1 (0.3)	1 (0.6)	0 (0.0)
Hispanic Ethnicity, N (%)			-			
Hispanic	4 (3.0)	0 (0.0)	-	10 (3.4)	4 (2.4)	0 (0)
Non-Hispanic	130 (97.0)	41 (100)	-	284 (95.3)	162 (97.6)	83 (98.8)
Unknown	0 (0.0)	0 (0.0)	-	4 (1.3)	0 (0.0)	1 (1.2)
Gender, N (%)			-			
Female	99 (73.9)	21 (51.2)	-	114 (38.3)	107 (64.5)	31 (36.9)
Male	35 (26.1)	20 (48.8)	-	184 (61.7	59 (35.5)	53 (63.1)
APOE4 Status, N (%)			-			
εX/ εX	100 (74.6)	39 (95.1)	-	146 (49.0)	66 (39.8)	30 (35.7)
εX/ε4 and ε4/ε4	34 (25.4)	2 (4.9)	-	152 (51.0)	100 (60.2)	54 (64.3)

* Other: ADNI = Asian; TARCC = Mixed race

### Measurement of Serum/Plasma Proteins

During clinical visits, a blood draw was collected from each subject; both plasma and serum were collected from TARCC subjects, who were non-fasting, whereas only plasma was collected from ADNI subjects, who were fasting. Plasma and serum were isolated from whole blood samples as described previously for each cohort[15]^,^ [18, 19]. Frozen specimens (serum for TARCC subjects and plasma for ADNI subjects), either from baseline or from the year-one follow-up exam, were shipped on dry ice to Myriad-Rules Based Medicine (www.rulesbasedmedicine.com, Austin, TX) where protein concentrations were assessed using a multiplex immunoassay panel for human analytes (human Multi-Analyte Profile, humanMAP). Samples were maintained in the frozen state until the time of the assay. Specifics regarding the sensitivity, specificity, range, inter-run variation coefficient, and spike recovery of the assays are available from Myriad-Rules Based Medicine.

The initial list of 11 proteins from the screening algorithm included: NRP, Beta 2 Microglobulin, C-Reactive Protein, Factor VII, Fatty Acid Binding Protein, I.309, Interleukin-18, Monocyte Chemotactic Protein 1, Pancreatic Polypeptide, Tenascin C, and Vascular Cell Adhesion Molecule 1. Proteins were removed from consideration if they failed to contribute to the O’Bryant et al. screening algorithm[12] in the same direction in both the TARCC vs. ADNI cohorts. Based on these criteria, C-Reactive Protein, Fatty Acid Binding Protein, I.309 and Interleukin-18 were excluded. The final list of seven proteins included Adiponectin, Beta 2 Microglobulin, Factor VII, Monocyte Chemotactic Protein 1, Pancreatic Polypeptide, Tenascin C, and Vascular Cell Adhesion Molecule 1.

### Genotyping

The TARCC cohort was genotyped using the Genome-Wide Human SNP Array 6.0 (Affymetrix, Santa Clara, CA), which includes 906,600 SNP markers. The ADNI cohort was genotyped using the Illumina 610-Quad BeadChip (Illumina, San Diego, CA), which includes 550,000 SNP markers. Both panels obtain genome-wide coverage. The BirdSeed v2 algorithm[20] was manually optimized and used for genotype calling.

### Quality Control Measures

Locally developed Java programs (collectively termed MACHTools) were used to perform critical data quality checking/filtering, imputation analysis, and data restructuring to affect overall computational performance. Participants were excluded from analysis if blood protein concentration data were not available, if the recorded sex did not agree with chromosome markers or if >5% of the markers did not successfully run. Markers were excluded from analysis if 5% of samples were missing, if they were monomorphic (threshold set at 0.01) or if they were out of Hardy-Weinberg Equilibrium (threshold set at 0.000001). In addition, genotype calls for important markers were manually checked independent of phenotype and recalled as necessary in order to account for obvious atypical hybridization intensities (such as discussed in (Didion et al.)[21][21] (21).This checking was conducted on the entire sample, without knowledge of diagnostic status or phenotype. Results were limited to loci with a minor allele frequency greater than 5%.

### Data Analysis

**Fig 1:**
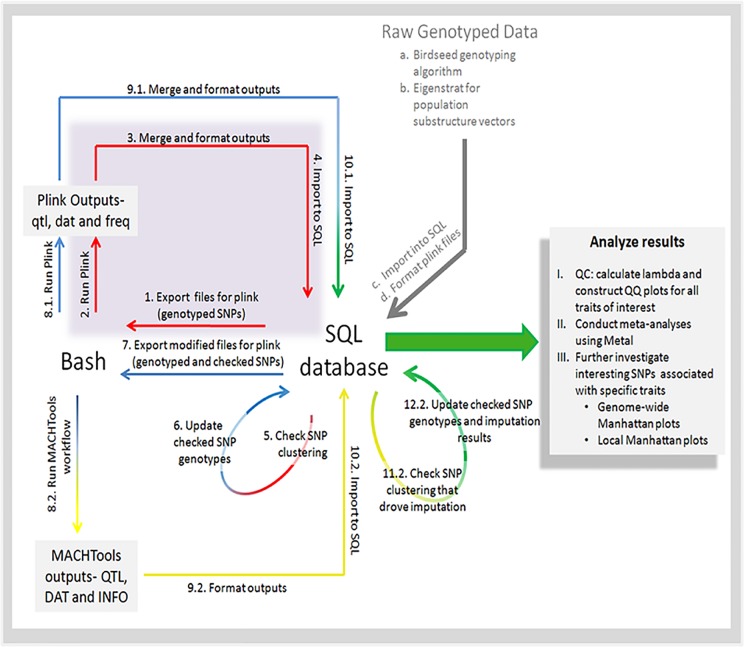
Data analysis workflow schematic.

An analysis pipeline was developed to fully analyze these GWA data in association with the quantitative RBM traits (Fig 1). Principle component analysis was performed using the Eigenstrat tool[22] for population substructure covariate determination. Relevant eigenvectors were used as covariates in the analyses, along with sex and education. The following plasma/serum protein concentrations were used as quantitative phenotypes: Adiponectin, Beta 2 Microglobulin, Factor VII, Monocyte Chemotactic Protein 1, Pancreatic Polypeptide, Tenascin C, and Vascular Cell Adhesion Molecule 1 (Table 2). Additionally, age-of-onset and case/control status were analyzed. Preliminary linear mixed model regressions were generated for all quantitative phenotypes using PLINK[23]. Phasing, imputation (using data from the HapMap II, HapMap III and 1000 Genomes databases), and subsequent regressions with the newly imputed GTs were performed using custom applications of the program MaCH[24]. Genotype calls at all significant SNPs were manually checked for proper clustering as described above. After re-clustering, the association regressions and imputation analyses were repeated. This iterative loop was executed three times. Lambda calculations and QQ plots were used to confirm the absence of underlying biases and/or confounders. Manhattan plots were generated for each GWA study in both TARCC and ADNI. Both Manhattan and QQ plots were generated using ggplot2 in R[25]. Final association results for typed and imputed SNPs in both TARCC and ADNI data sets were analyzed in conjunction using Metal[26]. For each quantitative trait, signals with p-values ≤1x10^-7^ in the meta-analysis were further investigated by plotting the local 1Mbp window (+/-500Kbp) for all three association studies (TARCC, ADNI, and Metal) using LocusZoom[27]. Only signals that were significant in the meta analysis at p ≤1x10^-7^ and showed evidence of a significant peak in both the TARCC and ADNI cohorts were reported.

**Table 2:** Serum protein measures.

	NC		MCI		AD	
Protein (Normalized Median (IQR))	TARCC N = 134	ADNI N = 41	TARCC N = 0	ADNI N = 298	TARCC N = 166	ADNI N = 84
Adiponectin	-0.2258 (-0.7516–0.5680)	-0.1664 (-1.3855–0.8740)	-	0.0759 (-0.6439–0.6987)	0.0851 (-0.5362–0.8213)	0.1991 (-0.5519–0.7738)
Beta 2 Microglobulin	-0.2030 (-0.7440–0.4490)	0.0130 (-0.6281–0.4855)	-	0.0130 (-0.8976–0.4855)	0.2170 (-0.5470–0.9100)	0.3422 (-0.3900–1.1211)
C-Reactive Protein	0.2795 (-0.5280–1.0170)	0.4301 (-0.2202–0.9687)	-	-0.0902 (-0.6694–0.5331)	-0.1870 (-0.8847–0.5227)	-0.0902 (-0.9172–0.8112)
Factor VII	0.0714 (-0.3993–0.6708)	0.3321 (-0.4641–1.0889)	-	0.0087 (-0.6516–0.4396)	-0.1235 (-0.6847–0.4562)	0.0914 (-0.6131–0.8496)
Fatty Acid Binding Protein	-0.1886 (-0.8014–0.4985)	0.1871 (-0.4197–0.9001)	-	-0.1360 (-0.6678–0.5627)	0.1847 (-0.6659–0.7723)	0.1581 (-0.4542–0.7569)
I.309	-0.0222 (-0.4888–0.5877)	0.0819 (-0.5711–0.9290)	-	-0.0442 (-0.6595–0.7106)	0.0470 (-0.5232–0.5967)	0.0002 (-0.7757–0.4838)
Interleukin-18	0.0303 (-0.5095–0.6723)	-0.2648 (-0.9204–0.8005)	-	-0.0881 (-0.6291–0.6027)	-0.2408 (-0.7006–0.4241)	0.0337 (-0.4914–0.5668)
Monocyte Chemotactic Protein 1	0.1898 (-0.3829–0.7083)	0.1413 (-0.3640–0.5187)	-	-0.0488 (-0.5593–0.4246)	-0.0610 (-0.6482–0.4144)	0.0730 (-0.3076–0.5375)
Pancreatic Polypeptide	-0.3350 (-0.9495–0.3015)	-0.3479 (-0.8845–0.2195)	-	-0.0968 (-0.6765–0.6208)	0.1050 (-0.4080–1.1152)	0.1147 (-0.5974–0.8456)
Tenascin C	-0.3015 (-0.8557–0.2412)	-0.5660 (-1.1650–0.2955)	-	-0.0150 (-0.7108–0.6640)	0.1450 (-0.4580–0.8990)	0.3060 (-0.5620–0.7600)
Vascular Cell Adhesion Molecule 1	-0.2350 (-0.9107–0.3712)	-0.0670 (-0.7370–0.5775)	-	-0.0670 (-0.6770–0.6565)	0.1420 (-0.4917–0.7665)	0.2470 (-0.4373–1.0150)

### Conditional Regression Methods

Relationships between diagnostic status, each protein biomarker and its associated genotypes were assessed in a series of conditional regressions. In this set of experiments, we included only combinations of proteins and genotypes that were identified as significant in the meta-analyses of both TARCC and ADNI cohorts (Table 3). The conditional regression analyses were conducted using a pair of analytical design models. The first analytical design was to use AD status (AD or NC) as the dependent variable, with either protein concentration or genotype as the independent variable. The resulting residuals from this regression were then used as the dependent variable in a second regression with either genotype or protein concentration as the independent variable. The second design was to use each protein concentration as the dependent variable with either AD status or genotype as the independent variable. The resulting residuals from this regression were then used as the dependent variable in a second regression with either genotype or AD status as the independent variable.

**Table 3:** Genome-wide significant signals for each endophenotype. P-values, chromosomal and gene location are presented for each signal from the meta-analyses (Meta) and from the individual (ADNI) and (TARCC) cohorts. P-values are also shown for the association between each endophenotypic signal and age of onset (AOO) and case-control status (CC).

Trait	Trait Chromosome	SNP(s)	P-value (TARCC)	P-value (ADNI)	P-value (Meta)	P-value (AOO)	P-value (CC)	SNP Chromosome	SNP Genes
ADN	3	chr3:186558403	9.56E-04	8.99E-06	3.16E-08	0.4903	0.4992	3	MAP3K13
		chr3:186562865	2.93E-04	2.94E-05	3.75E-08	0.4983	0.5105		
		rs57056768	7.40E-04	1.97E-05	5.53E-08	0.5121	0.5265		
		chr3:186552158	1.02E-02	2.85E-05	9.52E-07	0.5367	0.4756		
		rs8111139	1.79E-05	9.47E-04	2.21E-07	0.0026	0.9600	19	ZNF320
F7	13	rs561241	1.64E-02	7.96E-06	1.09E-08	0.4981	0.3392	13	F7
		rs3093233	1.60E-02	2.39E-04	1.78E-08	0.7954	0.2682		
		rs6039	1.63E-02	2.24E-04	1.87E-08	0.7902	0.2739		
		rs2480953	1.55E-02	1.97E-04	2.38E-08	0.8831	0.2627		
		rs9670535	1.57E-02	2.55E-04	2.67E-08	0.8743	0.2589		
		rs9669828	1.57E-02	2.49E-04	2.71E-08	0.8786	0.2652		
		rs9670502	1.57E-02	2.60E-04	3.03E-08	0.9689	0.9067		
		rs1046205	-	4.99E-08	4.99E-08	0.0141	0.5717		
		rs3093253	5.94E-05	6.67E-05	1.65E-07	0.5414	0.4311		
		rs569557	6.36E-06	1.46E-05	2.67E-07	0.5899	0.4263		
		rs2774033	3.71E-04	1.41E-05	2.75E-07	0.5621	0.4167		
		rs493833	3.96E-04	2.12E-05	4.13E-07	0.1443	0.6015		
		rs7327099	5.63E-04	1.64E-06	4.77E-07	0.5073	0.5070		
		rs6042	4.42E-04	1.76E-05	5.89E-07	0.9207	0.6075		
		rs11839532	-	7.86E-07	7.86E-07	0.4561	0.5065		
		rs6041	1.90E-02	9.03E-06	9.66E-07	0.0146	0.5644		
MCP-1	17	rs11663180	3.37E-05	1.03E-03	3.88E-07	0.5061	0.3007	18	ATP9B
		chr18:76959824	3.49E-05	1.02E-03	3.95E-07	0.5075	0.2990		
		rs10468812	1.21E-03	1.33E-04	4.87E-07	0.3812	0.4848		
		chr18:76954975	2.92E-05	1.51E-03	5.62E-07	0.4298	0.2323		
		rs60585035	2.74E-05	1.76E-03	6.49E-07	0.4572	0.2203		
		rs8085999	2.70E-04	4.51E-04	6.79E-07	0.3195	0.5519		
		chr18:76891698	4.05E-05	1.50E-03	6.99E-07	0.2722	0.1835		
		rs4324200	1.45E-04	7.18E-04	7.23E-07	0.1524	0.6783		
		rs59890467	3.54E-05	1.69E-03	7.37E-07	0.2713	0.1475		
		rs4471755	1.01E-03	1.58E-04	7.43E-07	0.4213	0.4191		
		rs57582689	1.49E-04	7.35E-04	7.54E-07	0.1474	0.6805		
		rs4799019	2.96E-05	1.92E-03	7.64E-07	0.4017	0.2107		
		rs1942306	1.88E-04	7.33E-04	7.79E-07	0.1474	0.6806		
		rs12607019	1.46E-04	7.33E-04	7.79E-07	0.1474	0.6806		
		rs1942308	1.56E-04	7.35E-04	7.80E-07	0.1475	0.6806		
		rs56894683	2.96E-05	1.96E-03	7.84E-07	0.3987	0.2088		
		rs57977665	2.97E-05	1.96E-03	7.87E-07	0.3989	0.2093		
		chr18:76891893	1.60E-04	7.33E-04	7.94E-07	0.1475	0.6806		
		rs9967354	1.61E-04	7.33E-04	8.00E-07	0.1475	0.6806		
		rs9966492	2.89E-05	2.02E-03	8.02E-07	0.3736	0.1910		
		chr18:76916979	5.83E-05	1.39E-03	8.21E-07	0.3959	0.1730		
		chr18:76895796	1.96E-04	7.27E-04	9.13E-07	0.1760	0.6762		
		rs9965326	6.55E-05	1.50E-03	9.70E-07	0.3597	0.1767		
		rs35548358	7.33E-02	1.05E-06	5.15E-07	0.1802	0.4824	6	TREM1
		rs34689624	7.35E-02	1.05E-06	5.17E-07	0.1799	0.4830		
		rs7761652	1.58E-01	6.05E-07	7.92E-07	0.1499	0.6995		

All conditional regressions were performed in R. The glm package was used for regressions when the dependent variable was continuous (protein concentration) and the lm package was used when the dependent variables were non-parametric (AD status or residuals). All initial regression equations were adjusted for sex, years of education and population substructure (10 most relevant Eigenvectors from the principal components analysis). For these analyses, an adjusted p-value of ≤0.01 in either cohort, or ≤0.05 in both cohorts was considered significant.

## Results

**Fig 2:**
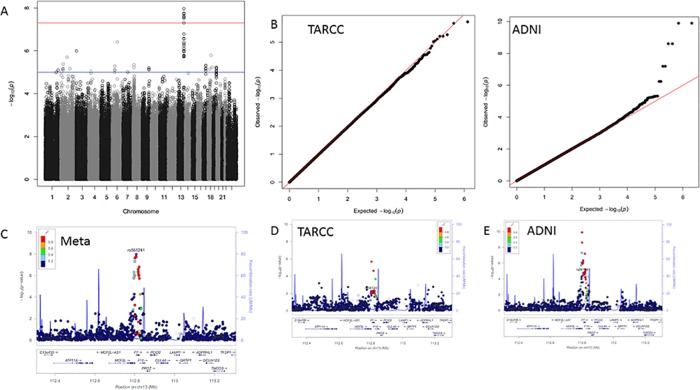
Factor VII results. Panel A; GWAS Manhattan plot for the meta-analysis of Factor VII. Panel B; QQ plots for the association results for TARCC (left) and ADNI (right). Panel C; LocusZoom plot for the chromosome 13 signal observed in the meta-analysis. Panels D and E; LocusZoom plots for the chromosome 13 signal in TARCC and ADNI, respectively.

**Fig 3:**
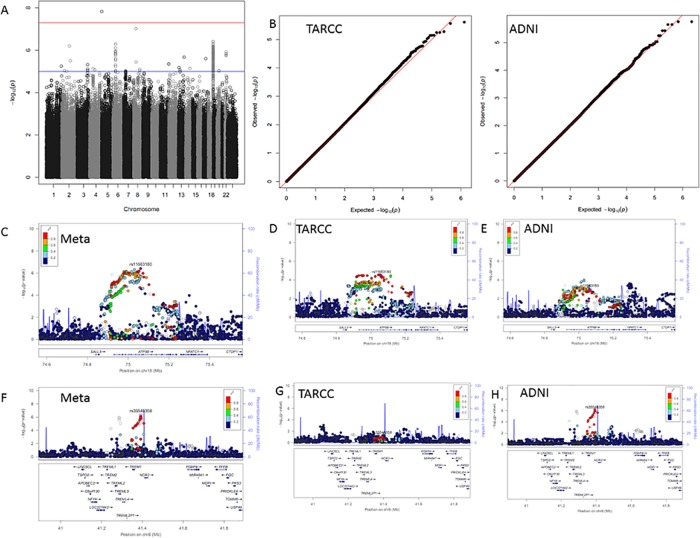
MCP1 results. Panel A; GWAS Manhattan plot for meta-analysis of MCP1. Panel B; QQ plots for the association results for TARCC (left) and ADNI (right). Panel C; LocusZoom plot for the chromosome 18 signal from the meta-analysis. Panels D and E; LocusZoom plots for the chromosome 18 signal in TARCC and ADNI, respectively. Panel F; LocusZoom plot for the chromosome 6 signal from the meta-analysis. Panels G and H; LocusZoom plots for the chromosome 6 signal in TARCC and ADNI, respectively.

**Fig 4:**
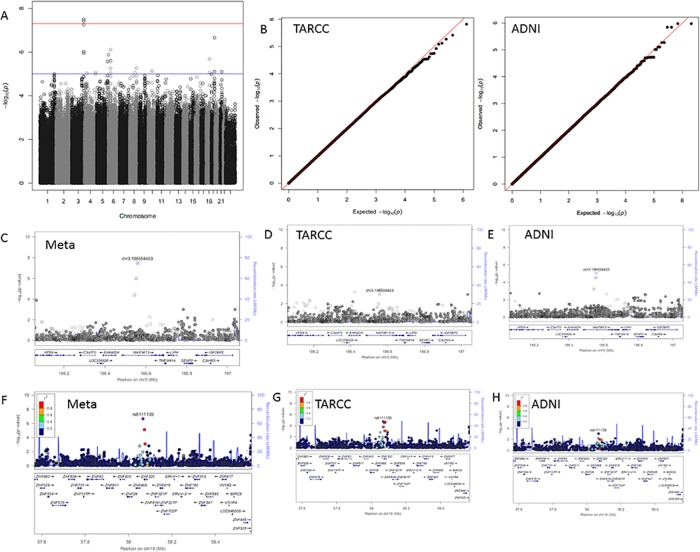
Adiponectin results. Panel A; GWAS Manhattan plot for meta-analysis of Adiponectin. Panel B; QQ plots for the association results for TARCC (left) and ADNI (right). Panel C; LocusZoom plot for the chromosome 3 signal from the meta-analysis. Panels D and E; LocusZoom plots for the chromosome 3 signal in TARCC and ADNI, respectively. Panel F; LocusZoom plot for the chromosome 19 signal from the meta-analysis. Panels G and H; LocusZoom plots for the chromosome 19 signal in TARCC and ADNI, respectively.

**Fig 5:**
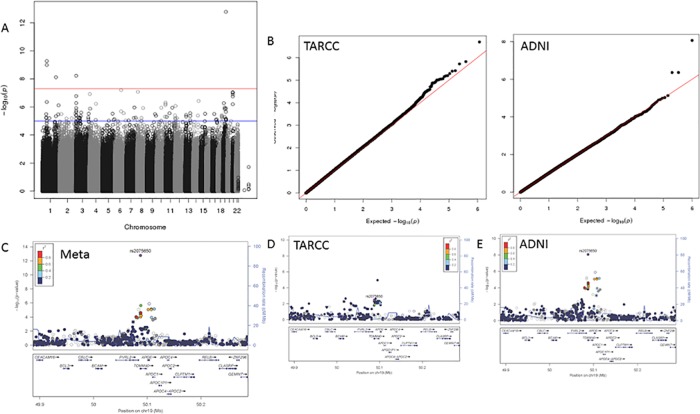
Case Control results. Panel A; GWAS Manhattan plot for Case Control status. Panel B; QQ plots for the association results for TARCC (left) and ADNI (right). Panel C; LocusZoom plot for the chromosome 19 signal from the meta-analysis. Panels D and E; LocusZoom plots for the chromosome 19 signal in TARCC and ADNI, respectively.

Quantile-quantile (QQ) plots showed no evidence of population substructure or inflation due to mistyped SNPs for any trait in either cohort (Figs 2–5). Meta-analyses showed many interesting signals (supplemental data), including a strong replication of the association between AD status and variants in the APOE/TOMM40 region. Conversely, no genome-wide significant (GWS; *p*<1x10^-7^) associations were observed for age of onset of disease symptoms. In this paper, we focus on four associations between genetic loci and three of the seven endophenotypes analyzed. In these four instances, the association reached GWS in the meta-analyses and evidence for each of the signals was observed in both the TARCC and ADNI cohorts (Table 3).

The strongest associations were found for blood concentrations of Factor VII (F7). This signal contained 12 SNPs on chromosome 13 within the F7 gene that were associated with serum/plasma concentrations of Factor VII at genome wide significance (Fig 2). Significance within this region ranged from *p* = 9.66x10^-7^ to *p* = 2.67x10^-8^. The signal within F7 is a sharp peak that roughly corresponds to the width of the F7 gene.

Seventeen SNPs on chromosome 18 were significantly associated with serum/plasma levels of monocyte chemoattractant protein -1 (MCP-1) (Fig 3). These polymorphisms were concentrated within the ATP9B gene. Significance within this signal ranged from *p* = 9.70x10^-7^ to *p* = 3.88x10^-7^. As with F7, the width of the ATP9B signal corresponds to the length and location of the ATP9B gene. Associations for many SNPs throughout the entire ATP9B gene region are elevated, forming a plateaued signal.

One of the many interesting associations that did not have support in both cohorts was between blood concentrations of MCP-1 and polymorphisms on chromosome 6 within the triggering receptor expressed on monocytes (TREM1) gene (Fig 3). In this case, the meta-analysis showed an association that reached genome wide significance (Table 3), but the signal was only apparent within the ADNI cohort (Fig 3).

Four SNPs on chromosome 3 were significantly associated with serum/plasma levels of adiponectin (Table 3). These polymorphisms were concentrated within the MAP3K13 gene. Significance within this signal ranged from *p* = 9.52x10^-7^ to *p* = 3.16x10^-8^. Unlike the signals within F7 and ATP9B, the width of the MAP3K13 signal for adiponectin is much narrower than the MAPK13 gene (Fig 4). In addition, although the signal reaches 10^−8^ for a pair of SNPs, there are far fewer SNPs in the MAP3K13 signal compared to the signals in F7 and ATP9B.

Despite the limited samples size in the present study, several previously reported associations for case control status were replicated at p≤0.05 and all published SNPs that were in the dataset showed a trend for association (Table 4). The most strongly associated SNP (rs2075650), which reached GWS for association with case control status (Fig 5) is located within the intron of the translocase of outer mitochondrial membrane 40 (TOMM40) gene[28]. TOMM40 is in the same region as the APOE gene, but has been reported to contribute additional genetic risk for AD[28].

**Table 4:** Associations between published AD SNPs and diagnostic status.

Gene	Marker	p-value
NME8	rs2718058	0.02
HKA DRB1-5	rs9271192	0.05
INPP5D	rs35349669	0.06
ZCWPW1	rs1476679	0.06
BIN1	rs6733839	0.07
CD2AP	rs10948363	0.07
EPHA1	rs11771145	0.08
CASS4	rs7274581	0.08
PTK2B	rs28834970	0.09
SORL1	rs11218343	0.09
ABCA7	rs4147929	0.09
CLU	rs9331896	0.09
MEF2C	rs190982	0.10
CR1	rs6656401	0.12
CLEF1	rs10838725	0.12
FERMT2	rs17125944	0.12
SLC24A4	rs10498633	0.12
PICALM	rs10792832	0.13
MS4A6A	rs983392	0.13

Conditional regression analyses recapitulated the GWAS results for F7, MCP-1 and adiponectin (Table 5). In addition, there were significant associations between AD status and blood concentrations of F7, MCP-1 and adiponectin, which were expected, given the membership of these proteins in the AD biomarker panel[12]. Finally, conditional regression analyses suggested that the SNPs contributed to variation in protein concentration independent of AD status. It was not possible to determine whether genotypes also contributed directly to disease risk.

**Table 5:** Results of conditional regression analyses. For each regression equation, dependent variables are listed in the top row, independent variables in the second row. For conditional regressions (two columns to the far right) the dependent variables were the residuals from an initial regression and the independent variables were the genotypes of candidate SNPs. [Protein~Disease status] indicates that protein concentration was the dependent variable and disease status was the independent variable in the initial regression. Similarly, [Disease status~ Protein] indicates that disease status was the dependent variable and protein concentration was the independent variable in the initial regression. Correlation statistics indicating the amount of variance explained by the independent variable is presented where appropriate for each regression. Results of regression analyses recapitulated the GWAS results for F7, MCP-1 and adiponectin (column one). In addition, there were significant associations between AD status and blood concentrations of F7, MCP-1 and adiponectin (column two). Conditional regression analyses suggested that candidate SNPs contributed to variation in protein concentration independent of AD status (column three). None of the results of regressions interrogating whether genotypes contributed directly to disease risk independent of protein endophenotype were significant (column four). However, due to insufficient statistical power, it was not possible to determine the true relationship between these factors.

		Dependent Variable		Protein concentration		Disease status		Residuals of Protein Concentration Regressed on Disease Status		Residuals of Disease Status Regressed on Protein Concentration	
		Independent Variable		Genotype		Protein concentration		Genotype		Genotype	
Cohort	Protein	Marker	Gene	p	r^2^	p	r^2^	p	r^2^	p	r^2^
TARCC	Adipo	chr3.186558403	MAP3K13	6.31E-10	24%	0.016	NA	0.001	4%	0.574	0%
ADNI	Adipo	chr3.186558403	MAP3K13	5.09E-05	31%	0.023	NA	0.017	5%	0.244	1%
TARCC	Adipo	rs8111139	ZNF320	3.91E-10	22%	0.026	NA	3.24E-04	4%	0.208	1%
ADNI	Adipo	rs8111139	ZNF320	3.58E-04	28%	0.023	NA	0.982	0%	0.653	0%
TARCC	F7	rs561241	F7	0.001	13%	0.001	NA	0.011	2%	0.386	0%
ADNI	F7	rs561241	F7	0.002	24%	0.069	NA	0.006	6%	0.998	0%
TARCC	MCP-1	rs11663180	ATP9B	0.022	9%	0.014	NA	3.03E-05	6%	0.959	0%
ADNI	MCP-1	rs11663180	ATP9B	0.010	21%	0.092	NA	0.003	7%	0.390	1%
TARCC	MCP-1	rs35548358	TREM1	0.759	3%	0.009	NA	0.054	1%	0.350	0%
ADNI	MCP-1	rs35548358	TREM1	0.031	19%	0.092	NA	0.012	5%	0.161	2%

None of the exact SNPs previously reported to be associated with Alzheimer’s disease were associated with blood concentrations of the proteins investigated in these experiments. However, signals were observed in the vicinity of CR1 (endoPT: Factor VII), MS4A6A/MS4A4E (endoPT: B2M), PICALM (endoPT: B2M and VCAM1), CLU (endoPT: Tenascin C), and PTK2B (endoPT: B2M).

## Discussion

The use of quantitative endophenotypes as outcome variables in genome-wide association studies has proven to be useful for identifying the genetic basis of complex disease[29–34]. This method is likely to be maximally effective for diseases that exhibit significant phenotypic heterogeneity, such as Alzheimer’s. The use of endophenotypes presumably provides increased statistical power due to greater proximity of the outcome variable to functional genetic variants, which reduces the impact of confounding non-genetic factors.

The strongest overall signal in the meta-analysis was between diagnostic status for Alzheimer’s disease and a group of SNPs in the region of the APOE gene. Given the well-replicated strength of the APOE signal, this result was not surprising even with the small sample size that was employed in the present study.

The strongest signal observed in the meta-analysis was between the serum/plasma concentration of F7 and a group of SNPs within the F7 gene on chromosome 13. Factor VII is a serine protease that is a key member of the coagulation cascade[35]. Along with tissue factor, F7 is responsible for initiating the coagulation cascade. The process begins with release of tissue factor from the external wall of blood vessels following vascular injury. Once inside the circulation, tissue factor binds to F7, which is converted to F7a, leading to conversion of factors IX and X into active proteases; factors IXa and Xa[35]. Factor VII is a vitamin K dependent enzyme and the target of warfarin and other anticoagulants that are used to prevent thrombosis and thromboembolism[36]. Serum concentrations of F7 were negatively associated with AD status in prior work[12].

Polymorphisms within the F7 gene have not been suggested previously as contributing to AD risk, despite multiple large-scale studies. Nevertheless, a SNP within this region (rs6046) has been associated with variation in risk for cardiovascular disease, venous thrombosis and stroke[37–41]; conditions that are associated with risk for AD and other forms of dementia. The rs6046 polymorphism, which is located in exon 9 and is predicted to cause the substitution of glutamine in place of arginine at amino acid position 353 (R353Q), has been shown to result in reduced levels of F7 activity[39]. The haplotype containing this SNP has been reported as both protective and a risk factor for coagulation related disease phenotypes[39–41]. The rs6046 SNP was associated with a later age of AD onset in our study.

Monocyte Chemoattractant Protein -1 (MCP-1) is one of the key chemokines involved in the regulation of monocyte and macrophage migration during the inflammatory response (see Deshmane et al. 2009 for review[42]). A variety of cells produce MCP-1, including epithelial, smooth muscle, astrocytes and microglial cells[42]. Increased MCP-1 has been shown to contribute to a variety of disease states, including Alzheimer’s disease,[43] atherosclerosis[44, 45], increased risk for AD following traumatic brain injury[46], insulin resistance[47], and neuronal death following ischemia[48]. Serum concentrations of MCP-1 were negatively associated with AD status in prior work[12].

We observed a significant signal in association with MCP-1 levels located on chromosome 18 in the coding region for ATP9B (ATPase, class II, type 9B). ATP9B is a class 2 P4-ATPase. Generally speaking, the P4-ATPases orchestrate phospholipid translocation from the exoplasmic to cytoplasmic leaflet which is critical for the maintenance of biological membrane characteristics and protein trafficking through vesicular transport. Alterations in the functionality of this family of flippases have been associated with multiple diseases and disorders (e.g., variants in ATP8B4 have been associated with Alzheimer’s disease[49, 50]). The ATP9B gene product has recently been shown to function independent of the CDC50 subunit complex, a characteristic unique to the class 2 P4-ATPases, and localize specifically to the trans-Golgi network[51]. The implied relationship between MCP-1 levels and the function of ATP9B gene products is not clear.

The adipocyte-derived hormone adiponectin (also known as 30-kDa adipocyte complement-related protein; Acrp30) has been mapped to a susceptibility locus for type 2 diabetes within the AdipoQ gene[52]. AdipoQ is shown to be dysregulated in obesity, metabolic syndrome, and cardiovascular disease[53–55]. Adipose tissues secrete many factors into the bloodstream, such as leptin, TNF-α, adipsin, and adiponectin. These proteins are referred to as adipocytokines, and are secreted to sensitize the tissues to insulin[52]. Levels of adiponectin in the blood are lower in individuals with diabetes, insulin resistance, and obesity.[56] Serum concentrations of adiponectin were positively associated with AD status in prior work[12].

We observed two significant signals in association with adiponectin levels. The first signal is located on chromosome 3 within the MAP3K13 gene. The MAP3K13 gene encodes a protein kinase that is expressed most strongly in the pancreas, brain, and liver, but not detected in heart, lung, skeletal muscle, or kidney. Protein kinases are involved in a litany of pathways, however MAP3K13 has been shown to be involved in the stress-activated JNK1 pathway[57]. This gene’s specific involvement with adiponectin expression is unclear.

The second signal associated with adiponectin levels was located on chromosome 19 within a zinc finger gene, ZNF320. Zinc fingers are a heterogeneous class of protein structural motifs that are involved in the expression or repression of genes. Specifically, ZNF320 has been shown to be implicated in glioblastoma. The ZNF320 gene’s involvement with adiponectin expression is also unclear.

Results of the current study appear to confirm the utility of an endophenotypic approach to the analysis of GWAS data from complex traits. Four novel associations were observed between genetic variants and endophenotypes for AD. Polymorphisms within the F7, ATP9B, MAP3K13 and ZNF320 genes have not been suggested previously as contributing to AD risk, despite multiple large-scale GWAS studies[1, 58–65]. Interestingly, meta-analysis showed that two SNPs within the F7 gene (including rs6046) showed a trend for association with age at disease onset in our sample (Table 3). Unsurprisingly due to the relatively small sample, none of the SNPs were associated with diagnostic status.

In an attempt to determine the specifics of the most likely biological relationship model for AD, a series of conditional regressions were performed that assessed the relationships between diagnostic status and non-genetic factors as well as specific protein biomarkers and their associated genotypes. The majority of significant associations recapitulated either current GWAS relationships between specific SNPs and blood protein concentrations, or relationships inherent to the AD biomarker panel between blood protein concentrations and disease status. In addition, associated genotypes explained a significant amount of the variation that remained in protein concentration after disease status and covariates (sex, population substructure eigenvectors) were accounted for. It was not possible to determine conclusively whether associated SNPs also contributed directly to AD risk, independent of protein concentration.

It was interesting that several AD loci that have been reliably reported in the literature were associated with our AD endophenotypes. If these associations are confirmed in independent cohorts, they may help to explain the etiological mechanisms and functional variants that are responsible for these previously published associations.

There a number of caveats to these results. First, although the observations reported were derived from a meta-analysis of two entirely independent cohorts of study participants, the sample sizes were small. This is particularly true in light of recent publications by the IGAP group, which were based upon an international sample of nearly 75,000 individuals. However, the analytical approach that we adopted provided much greater statistical power than would have been possible with a traditional GWAS, where diagnostic status is used as the outcome variable.

In summary, the use of endophenotypes for Alzheimer’s disease in the place of diagnostic status as the outcome variable in GWAS analysis overcame sample size constraints and allowed the identification and independent replication of two putative novel genetic loci that appear to impact risk for AD. Polymorphisms in F7 and ATP9B may impact the risk or development of AD and should be studied in a larger, independent cohort.
